# Radiologist burnout: AI’s true black box

**DOI:** 10.1007/s00330-025-12278-6

**Published:** 2026-01-16

**Authors:** Jay R. Parikh, Frank J. Lexa

**Affiliations:** 1 Division of Diagnostic Imaging, The University of Texas MD Anderson Cancer Center, Houston, TX USA; 2 Radiology Leadership Institute of the American College of Radiology, Reston, VA USA

**Keywords:** Artificial intelligence, Radiology, Burnout, Wellness, Radiologist

## Abstract

**Abstract:**

Multiple articles have touted the longitudinal promise of artificial intelligence (AI) in radiology, including projections of streamlining repetitive tasks, improving workflow, and reducing physician burnout. The purpose of this article is to review publications directly assessing the impact of AI on radiologist burnout and the impact of AI on the established drivers of radiologist burnout. Our analysis found conflicting, inconclusive limited data that AI reduces radiologist burnout, and the balance of data does not support that AI improves the drivers of burnout. How AI affects radiologist burnout remains a “black box”, with the final impact yet to be determined.

**Key Points:**

***Question*** *While AI has been touted to reduce radiologist burnout, the literature to date supporting this claim has not been explored*.

***Findings*** *Our analysis found inconclusive, limited data that AI reduces radiologist burnout, and that the balance of data does not support that AI improves the drivers of burnout*.

***Clinical relevance*** *Despite the optimism towards AI implementation in radiology, how AI truly affects radiologist burnout remains a “black box”, with the final impact yet to be determined*.

## Introduction

According to the World Health Organization, burnout is a syndrome resulting from chronic workplace stress that has not been successfully managed [1]. Burnout is characterized by three dimensions- feelings of exhaustion or energy depletion, negativity or cynicism towards one’s job, and reduced professional efficacy [2]. Multiple peer-reviewed publications have consistently demonstrated a high prevalence of radiologist burnout across multiple radiology sub-specialties [3–7], practice settings [8, 9], and leadership roles [10, 11]. Physician burnout has been linked with multiple adverse metrics [12]. In one systematic review and meta-analysis, physician burnout was associated with a nearly four times decrease in job satisfaction, career choice regret, by more than threefold, turnover intention by over threefold and a doubling of patient safety incidents and low professionalism [13].

AI can be conceptualized as the development and implementation of computer systems capable of performing tasks that traditionally required human levels of intelligence [14]. Complex calculations are carried out by machines to not only learn but also adapt to the reading environment to generate outputs available to radiologists. These AI systems, however, are very much a “ black box” for radiologists, who do not know how the machine learning systems come to develop the results that they see portrayed on their images [15–17].

Several articles and manufacturers have touted the longitudinal promise of artificial intelligence (AI) in radiology, including projections of streamlining repetitive tasks, improving workflow, and reducing physician burnout [14, 18]. A recent survey of chairs of academic departments within the United States found 80 percent optimistic for AI, with the two dominant reasons for planned implementation being improved quality and efficiency, and reducing radiologist burnout [19].

For AI to overcome known radiologist skepticism and to increase the adoption of the technology, AI implementations will need to scientifically demonstrate reduced radiologist burnout. The purposes of this article are to describe publications directly assessing the impact of AI on radiologist burnout, and to review publications of AI affecting the established drivers of radiologist burnout.

## Studies of AI and radiologist burnout

Very few randomized controlled trials have been published in the AI space [20]. Specifically, the majority of publications studying the impact of AI on radiologists are review articles [15]. Two recent peer-reviewed publications evaluated the association of the use of AI with radiologist burnout using validated items. Both studies have been carried out in China, where there has been widespread adoption of AI.

The first investigation by Liu et al [21] was a national cross-sectional study of 6726 radiologists, of which 3017 were in the AI group. The weighted prevalence of burnout was significantly higher in the AI group compared with the benign AI group (40.9% vs 38.6%; *p* < 0.001). The use of AI was associated with radiologist burnout, demonstrating a dose–response association. Joint exposure to AI use alongside either high workload or low AI acceptance was associated with an additional risk of burnout.

The second study by Fang et al [22] deployed a cross-sectional research design conducted amongst radiologists and technologists from 68 public hospitals and 63 cities in 20 provincial-level administrative regions of China. The majority of survey respondents (66.1%) were radiologists. While burnout amongst radiology technicians was slightly higher than radiologists, radiologists had a greater proportion of moderate to severe burnout than technologists. The duration of AI use amongst radiologists was significantly negatively correlated with burnout. The authors go on to conclude that encouraging the use of AI software and radiology can be used to help prevent and alleviate occupational burnout.

At first glance, the two studies have diametrically opposite conclusions regarding the association of AI and radiologist burnout. Part of this may be attributed to the length of AI usage in the two study cohorts. Both studies used a Chinese version of the Maslach Burnout Inventory-Human Services Survey (MBI-HSS) to assess burnout. The MBI-HSS has been validated in the United States; validation in China is not as robust. This may have also contributed to variation in results. Moreover, it is unknown how much the work environment for radiologists in China overlaps with the work environment within the United States and other countries. Specifically, further studies need to be done on cultural expectations regarding the quality of interpretation, tolerance for error, and the consequences of errors, including malpractice and other factors. Therefore, definitive conclusions cannot be reached at this time from the studies on the impact of AI on radiologist burnout.

## Studies of AI and the drivers of radiologist burnout

With limited studies available regarding the direct impact of AI on radiologist burnout, to help expand the analysis of the potential impact of AI on radiologist burnout, we also evaluated studies of AI on the established drivers of burnout. Previous work has identified six primary drivers of physician burnout, assuming a core purpose of meaning at work [23]. These drivers are workload and job demands, control and flexibility, work-life integration, social support and community at work, organizational culture and values, and efficiency and resources (Table 1).

**Table 1:** Drivers of burnout in radiologists^a^

Driver of burnout	Key contributing factors
Workload and job demands	High productivity expectations Compensation linked to volumes Uneven distribution of caseload
Control and flexibility	Lack of autonomy in managing clinical Workflow Inflexible work hours Unsupported vacation time
Work-life integration	Extended work hours Work responsibilities continued at home Added call responsibilities
Social support and community at work	Isolation from peers Lack of collegiality amongst peers Lack of social gatherings
Organizational culture and values	Poor leadership behavior Lack of equity Lack of fairness
Efficiency and resources	Operational inefficiencies Unnecessary interruptions Lack of support staff

^a^ Adapted from Shanafelt et al [23], assuming a radiologist has a core purpose at work

### Work and job demands

Radiologist workload - the product of the number, size, and complexity of examinations performed per time unit [24] - has increased dramatically over the past two decades [25–27]. In the context of declining reimbursement, there has been increased pressure to raise relative value unit (RVU) productivity to maintain income. Higher workloads have been consistently identified as a stressor risk factor for radiologist burnout [4, 28, 29]. The stress level has been heightened by rising caseloads of urgent examinations that interrupt workflow and have high-intensity levels, such as acute stroke and trauma studies.

AI has been proposed as a solution to address radiologist workload with the potential to improve clinical outcomes [30]. In mammography, AI has been proposed in some studies to be able to replace and even outperform radiologists as a stand-alone reader, offloading normal screening examinations [31–33]. In the screening environment, workflows are different than the diagnostic settings [34, 35]. In an uninterrupted setting, radiologists have the ability during screening to participate in high-volume, efficient batch interpretation [36]. The high volume of normal studies allows the radiologist to recalibrate and remember normal anatomy. Active participation in screening also has implications for teaching, as radiologists would like to be able to teach the next generation how to interpret normal studies. Additionally, since work RVU is often coupled to salary in many practice environments within the United States, this does not consider case complexity, which could negatively impact radiologist salary [37]. Lastly, radiologists solely focusing all day on only highly complex studies, such as post-treatment oncology body studies or post-operative brain tumors, may cause stress and negatively impact their wellness.

In a survey of 675 members of the European Society of Radiology, there was a split in the perceived expected impact of total reporting workload with AI, with 50.8% expecting a reduced reporting workload, while 49.2% expected an increased workload [38]. A study of a random sample of 440 medical imaging studies published in 2019 found that while they added value to patient care, they increased the workload of diagnostic radiologists, especially as it applied to AI studies [24]. In multivariate analysis, only AI as primary research area remained significantly associated with an increased workload (*p* < 0.001, with an odds ratio of 10.64).  Increased workload attributed to AI is at least partially from elevated processing and interpretation times [21, 24].

Several studies have demonstrated that workload limits are needed to reduce medical errors and increase patient safety [39–41]. If, despite all efforts, the workload is still above the limits of a radiologist, then the use of AI would translate into a delay of the problem rather than a solution [15].

### Control and flexibility

Stand-alone image interpretation by AI has been proposed [42]. In this circumstance, the radiologists will need to feel comfortable relinquishing complete autonomy to the AI platform. This may lead to moral injury, as the radiologist may realize the imperfections of AI, and grapple with both false negative, as well as false positive outcomes.

Medicolegal liability is a well-established concern for radiologists in the modern culture of medicine. For example, in a survey of stressors affecting radiologists within the Society of Breast Imaging, fear of a lawsuit was the third most common stressor, affecting over 52% of respondents [28]. A survey of neuroradiologists in the ASNR showed that medicolegal entanglements were common and contributed to burnout [43].

Relinquishing partial or complete autonomy of image interpretation to AI is coupled with increased liability risk from the AI missing a lesion. Current AI algorithms struggle with suboptimal data inputs, atypical patterns, and rare lesions on imaging [14]. In the survey of the European Society of Radiology, 41% of responders foresaw the radiologist taking the legal responsibility of an adverse AI systems outcome, while 41% of responders favored shared responsibility by the radiologist and the manufacturer [38].

AI can also create false positives, creating a further source of stress for radiologists. In some institutions, permanent records of AI findings are kept on images in PACS. Recent data suggest that perceived legal culpability for radiologists will be higher when they miss an abnormality that is detected by AI [44]. Some radiologists, when they disagree with the AI, now incorporate a statement in their reports as to why they are countering the AI's false positives. This practice can slow down the workflow of the radiologist and reduce productivity while creating resentment and burnout from added low-value activities.

### Work-life integration

Currently, there is no robust longitudinal data regarding the impact of AI on radiologists' life balance.

Lack of work-life balance is an established stressor for radiologists that contributes to radiologist burnout [28, 29]. If AI can reduce workload for radiologists without reducing salary, it would reduce radiologist stress. Many radiologists currently practice remotely and favor remote reading to improve work-life balance [45]. AI-powered tools can facilitate remote reading of images [46], enabling radiologists to work from different locations and potentially improve their work-life balance by avoiding long commutes. By handling more routine tasks during regular hours, AI could potentially minimize the need for radiologists to work late or on call, leading to a better work-life balance.

Longitudinally, AI may also compromise the work-life integration of radiologists. With an increased number of AI apps in the work environment, there is potential for information overload and consumption of radiologist time [14]. The increased workload could worsen the current trend of blurring personal and work lives as radiologists allow themselves to be reached after hours at work, both from the worksite and also from home. Many hospital administrators and radiology practice leaders now leverage remote capabilities to make radiologists available all the time, especially when there is remote access to the electronic medical record. Radiologists need downtime for digital detoxification and to recharge their batteries [47].

### Social support and community at work

Following PACS, radiologists have become increasingly isolated from both patients and referring clinicians[48]. Loneliness and social isolation have been associated with both short- and long-term adverse outcomes, including decreased impulse control, sleep fragmentation, depression, cognitive decline, and dementia [49–52]. Social isolation is associated with increased burnout and suicidal ideation, and is more common among US physicians than workers in other fields [53]. Peer-reviewed studies have demonstrated that restoring a sense of community can help reduce physician burnout [54, 55].

The long-term impact of AI on social support and commensality are currently unknown. It can be argued that the increased time struggling with AI algorithms may translate into radiologists spending more time at their workstations, further increasing isolation.

On the other hand, if AI does in fact translate into reduced workload, then there would be increased time for radiologists to theoretically socialize both at work and outside of work. Furthermore, similar to radiology societies that have collaborated on AI [56], AI-centric communities may ultimately develop within individual practices in which like-minded individuals using similar technologies may want to socialize, collaborate, and innovate.

### Organizational culture and values

With practice consolidations over the past decade, many radiologists practice within larger organizations [57]. Within organizations, previous work has demonstrated that alignment of physicians' personal and organizational values is inversely correlated with physician burnout [58] and that physicians experience their organization through the prism of their work unit leader [59]. Leadership is inversely related to physician burnout [58], and therefore leaders have critical roles to play with regard to burnout in radiologists, both for good and for ill [60].

Currently, AI in radiology is still relatively early in the adoption cycle, and the timing is very important. Leaders who want to promote effective use of AI in their departments can deploy several strategies. First, they must choose systems carefully with a radiology-centric vision [61]. AI programs that are written for non-radiologists may add little or no value in the reading room. Second, radiologists need to provide feedback to leaders who have the courage to jettison systems and/or replace programs that aren’t adding value [62]. Third, leadership is local and therefore leaders need to measure and manage burnout in their practices [63]. Leaders need to objectively assess and understand if the newly implemented AI is making the lives of their radiologists easier or slowing them down and/or burning them out. Fourth, leaders need to remember that we are in a labor crisis with a shortage of radiologists and pre-existing burnout [64, 65]. Value to radiologists today must outweigh any associated negative elements in AI usage. Leaders who use AI to the advantage of their radiologists will do better with recruitment and retention.

### Efficiency and resources

The random sample of medical studies published in 2019 found that in non-academic general teaching hospital settings, the primary cause of increased workload was increased interpretation time, often combined with increased post-processing and acquisition time [24].

A recent systematic literature review of the effects of AI implementation on efficiency in medical imaging identified 48 original studies [66]. The three studies that were randomized were appraised with an overall high risk of bias. Additionally, 19 studies declared a relevant conflict of interest, and six other studies had potential conflicts of interest, adding up to more than 50% of the included studies. Although most studies reported positive effects, three separate meta-analyses of 12 studies did not show significant effects after AI implementation. While almost 67% of time-related outcome studies showed a decrease in time with AI usage, the meta-analysis with subsets of comparable studies showed no evidence of AI tools reducing the overall time on imaging tasks. The authors point out that a noteworthy portion of these studies reveal conflicts of interest, potentially influencing study design and outcome estimation.

The balance of data thus far has therefore not conclusively demonstrated that AI has improved radiologist efficiency. Additionally, efficiency can only be maximized to a finite extent. Radiologists can run faster and faster on a treadmill before they collapse. There are also downsides to efficiency. Some experts argue that the antidote to burnout is slack [67]. Creativity and innovation occur during slack, especially important in the academic setting [68].

## Summary

Our analysis found conflicting, inconclusive limited data that AI reduces radiologist burnout, and the balance of data does not support that AI improves the drivers of burnout. The term “black box” has been accepted for decades in the literature to explain how radiologists do not understand, within AI, the hidden layers of deep learning. Despite the optimism towards AI implementation in radiology, how AI affects radiologist burnout remains a “black box”, with the final impact yet to be determined. More primary investigations are needed to help unveil the black box and help radiologists understand the true impact of this evolving technology on their overall already compromised wellness.

## Abbreviations

AI: Artificial intelligence
MBI-HSS: Maslach burnout inventory-human services survey
RVU: Relative value unit
